# Effect of sodium/calcium hypochlorite on adhesion and adaptation of fiber posts luted with a dual resin cement

**DOI:** 10.1590/0103-6440202305396

**Published:** 2023-07-17

**Authors:** Guilherme Pauletto, Israel Bangel Carlotto, Lucas Saldanha Da Rosa, Gabriel Kalil Rocha Pereira, Carlos Alexandre Souza Bier

**Affiliations:** 1 Post-Graduate Program in Oral Science, Faculty of Dentistry, Federal University of Santa Maria (UFSM), Santa Maria, Rio Grande do Sul State, Brazil

**Keywords:** bond strength, calcium hypochlorite, sodium hypochlorite, glass fiber post, resin cement

## Abstract

This study aimed to evaluate the effect of different concentrations of sodium hypochlorite (NaOCl) and calcium hypochlorite [Ca(OCl)_2_] on the bond strength and adaptation of glass fiber posts luted with a dual-curing resin cement. Fifty decoronated premolars were sectioned 14 mm from the apex and endodontically treated. The root canal filling was partially removed. The specimens were divided into 5 groups (n=10) according to the irrigant for post space irrigation: 0.9% sodium chloride (NaCl), (control); 2.5% NaOCl; 5.25% NaOCl; 2.5% Ca(OCl)_2_; and 5.25% Ca(OCl)_2_. For each group, irrigation was performed with 5 ml of solution. Afterward, the posts were luted with a dual-curing resin cement. One slice from each third was obtained and submitted to the push-out test and failure modes analysis. An additional slice from the middle third was submitted to confocal images for analysis of adaptation failures (gaps). Two-way ANOVA, Tukey’s post-hoc, Kruskal-Wallis with Bonferroni adjusted, and chi-square tests, analyzed data. The group treated with 5.25% NaOCl showed lower bond strength values and generated more cohesive failures compared to the control (p < 0.05). Bond strength decreased from coronal to apical in the post space (p < 0.001). The groups treated with NaOCl had the highest percentages of gaps compared to the control (p < 0.05). Regardless of concentration, Ca(OCl)_2_ did not influence the bond strength and the occurrence of gaps (P > 0.05). Ca(OCl)_2_ is a good option for irrigating the post space before luting a fiber post with a dual-curing resin cement.

## Resumo

Este estudo teve como objetivo avaliar o efeito de diferentes concentrações de hipoclorito de sódio (NaOCl) e hipoclorito de cálcio [Ca(OCl)_2_] na resistência de união e adaptação de pinos de fibra de vidro cimentados com um cimento resinoso dual. Cinquenta pré-molares tiveram suas coroas removidas, foram seccionados a 14 mm do ápice e tratados endodonticamente. A obturação do canal radicular foi parcialmente removida. Os espécimes foram divididos em 5 grupos (n=10) de acordo com o irrigante para irrigação do espaço do pino: cloreto de sódio 0.9% (NaCl), (controle); NaOCl 2.5%; NaOCl 5.25%; Ca(OCl)_2_ 2.5%; e Ca(OCl)_2_ 5.25%. Para cada grupo, a irrigação foi realizada com 5 ml da solução. Posteriormente, os pinos foram cimentados com um cimento resinoso dual. Uma fatia de cada terço foi obtida e submetida ao teste push-out e análise dos modos de falha. Um corte adicional do terço médio foi submetido a imagens confocais para análise de falhas de adaptação (*gaps*). Os dados foram analisados pelos testes ANOVA de duas vias, post-hoc de Tukey, Kruskal-Wallis com ajuste de Bonferroni e qui-quadrado. O grupo tratado com NaOCl 5.25% apresentou menores valores de resistência de união e gerou mais falhas coesivas em relação ao controle (p < 0.05). A resistência de união diminuiu de coronal para apical no espaço do pino (p < 0.001). Os grupos tratados com NaOCl apresentaram os maiores percentuais de *gaps* em relação ao controle (p < 0.05). Independentemente da concentração, o Ca(OCl)_2_ não influenciou a resistência de união e a ocorrência de *gaps* (P > 0.05). O Ca(OCl)_2_ é uma boa opção para irrigar o espaço do pino antes de cimentar um pino de fibra com um cimento resinoso dual.

## Introduction

The longevity of a restorative procedure is directly related to the amount of remaining tooth structure and the effectiveness of the adhesive protocol ^1^. Endodontically treated teeth, that show extensive coronary destruction, often require root retention for coronary reconstruction using an intraradicular post ^(^ ^2^. Glass fiber posts are recommended for devitalized teeth, as they have a similar elastic modulus to dentin, are aesthetically superior, and are cost-effective ^(^ ^3^.

Adequate bonding between cement and dentin is important for post-retention ^1^. The post space is vulnerable to microbial contamination, recommending the use of irrigates similar to those used in endodontic treatment ^(^ ^4^ However, endodontic irrigants can negatively affect the bonding procedure ^(^ ^5^. Sodium hypochlorite (NaOCl) is the most widely used solution in Endodontics due to its antimicrobial action and tissue dissolution capacity ^(^ ^6^. However, NaOCl is associated with a significant deproteinization of dentin ^(^ ^7^ and worse bond strength values in the adhesive luting of fiber posts ^(^ ^5^. Based on that, there is still no consensus on the ideal solution for use in the post space, as the irrigant must present a balance between its antimicrobial action and its influence on dentin adhesion ^8^. Recently, promising investigations have emerged regarding the use of calcium hypochlorite [Ca(OCl)_2_] in Endodontics ^(^ ^9^ ^,^ ^10^. Ca(OCl)_2_ has similar antimicrobial action to NaOCl ^(^ ^9^ and seems to induce less structural changes to dentin ^(^ ^11^.

Despite the aforementioned presupposes, the influence of Ca(OCl)_2_ on the adhesion of fiber posts was the subject of scarce studies, with conflicting results ^(^ ^12^ ^,^ ^13^ ^,^ ^14^. Furthermore, none of these studies has compared the influence of Ca(OCl)_2_ on the adaptation of resin cement to dentin in the post space.

Therefore, the purpose of this in vitro study was to evaluate the bond strength in the different dentinal thirds of the post space and adaptation failures (gaps) at the cement/dentin interface, resulting from the interaction between dual-curing resin cement and a substrate irrigated with different concentrations of NaOCl or Ca(OCl)_2_. The study adopted null hypotheses was are that there would be no difference in bond strength from the use of different irrigants, regardless of the dentinal third (I); and that the different solutions and concentrations would also not have a significant effect on the presence of gaps (II).

## Materials and methods

### Ethical approval and sample selection

The ethics committee (no. 50355021.0.0000.5346) approved this research. The sample size was calculated (G*Power; Heinrich-Heine-Universität, Düsseldorf, Germany), considering: power = 90%, alpha-type error = 0.05 and effect size = 0.59 ^(^ ^15^. A total of 50 specimens (ten teeth per group) were indicated as the ideal size. Fifty permanent human premolars were used. Digital periapical radiographs were performed to select single-rooted teeth, with a single main canal and complete root development, free of root caries, previous endodontic treatment, calcifications, resorption, and cracks/fractures. The teeth were kept in 1% chloramine-T solution for 48 hours and then stored in distilled water at 4ºC until the following methodological steps.

### Sample preparation

The teeth were decoronated with a diamond disc under constant irrigation in a precision cutting machine set at 300 rpm (Isomet 1000; Buehler Ltd, Lake Bluff, USA). The roots were standardized at 14 mm and the working length was established at 1 mm from the apical foramen. The root canal was prepared with the Bassi Logic rotary files (Easy Equipamentos Odontológicos, Belo Horizonte, MG, Brazil), sizes 40.01 and 40.05, under 20 mL 2.5% NaOCl irrigation. After instrumentation, we used 2 mL of 17% EDTA for 1 minute, repeated 3 times, and a final irrigation with 10 mL of distilled water was performed. Then, the root canals were dried with 40.05 absorbent paper points (Easy Equipamentos Odontológicos). All roots were filled using the lateral condensation technique with gutta-percha and AH Plus sealer (Dentsply Maillefer, Ballaigues, Switzerland). Digital periapical radiographs were performed to confirm the quality of the filling. The roots were sealed using interim restorative material (Coltosol; Coltene, Alstatten, Switzerland) and stored in distilled water at 37ºC for one week.

The coronary seal was removed and the gutta-percha was then partially removed using 2 and 3 Largo burs (Dentsply Mailleifer), keeping the final 4 mm. Digital periapical radiographs were performed to confirm the desobturation. The post space was prepared using the Exacto Translúcido Angelus N2 bur (Angelus, Londrina, PR, Brazil). The samples were randomly allocated (http://www.randomized.org), according to the irrigant used, in five groups (n=10): 0.9% sodium chloride (NaCl), (control); 2.5% NaOCl; 5.25% NaOCl; 2.5%Ca(OCl)_2_; and 5.25% Ca(OCl)_2_. The post space was irrigated for 60 seconds with 5 mL of the irrigant and held in place for 3 minutes without agitation. Subsequently, the root canals were dried with 40.05 absorbent paper points (Easy Equipamentos Odontológicos).

The glass fiber posts (Exacto Translucido Angelus N2; Angelus) were cleaned with 70% alcohol, coated with silane (Monobond N; Ivoclar, Schaan, Liechtenstein), allowed to rest for 1 minute and air-dried. The post space was treated with primers (Multilink N Primer A + Multilink N Primer B; Ivoclar) for 30 seconds, the post was covered with resin cement (Multilink N, Ivoclar) (Box 1) and then inserted into the conduit up to the established length with rotary movements, and the set was light-cured for 40 seconds using a light-curing unit (Radii Cal; SDI, Bayswater, Australia) operating at 1200 mW/cm^2^. The specimens were stored in distilled water at 37ºC during one week.

### Push-out assessment

The samples were sectioned using a precision cutting machine (Isomet 1000; Buehler) set at 300 rpm and equipped with a diamond disc, obtaining four slices per sample, with a thickness of 1mm ± 0.1mm (one slice from the cervical and apical thirds, and two slices from the middle third).

**Box 1:**
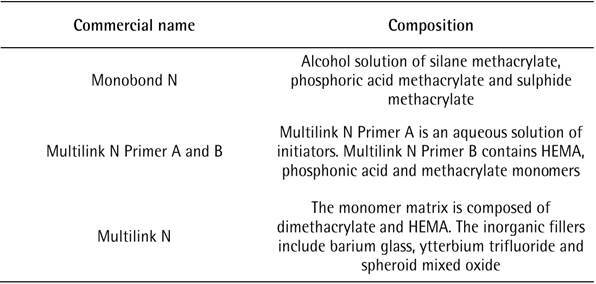
General description of the dual-curing self-etching resin cement system used (Ivoclar Vivadent)

A slice of each third was positioned in a universal testing machine (Emic DL-2000; Emic, Pinhais, PR, Brazil). The post was loaded in the apical-coronal direction using a stainless-steel plunger (Ø = 0.8 mm) at a speed of 0.5 mm/min until failure. The data obtained in newtons (N) were converted into megapascals (MPa) using the following formula: σ = F / A. F is the force for specimen rupture (N), and A is the bond area (mm^2^). The following formula was used to determine the bonded interface area:

$$A=\;\pi (R+r)\sqrt{h^{2}+(R-r)²}$$

In the formula, π = are the constant 3.14, R = coronal radius, r = apical radius, and h = slice thickness ^(^ ^16^. A digital caliper was used to obtain measurements (CD-15C; Mitutoyo Co., Kawasaki, Japan).

### Failure modes analysis

After failure, a blinded examiner, through a stereomicroscope (Discovery V20; Carl-Zeiss, Gottingen, Germany) at × 40 magnification, evaluated the samples. Failure patterns were classified into: Ac/d = Predominant adhesive at cement/dentin interface; Ac/p = Predominant adhesive at cement/post interface; C = Cohesive of dentin. The calibration consisted of repeating the analysis of the fracture pattern of 30 slices, with an interval of two weeks. Examiner reproducibility, which was calculated using the Kappa test, was 0.943.

### Evaluation of adaptation in cement/dentin interface

**Figure 1:**
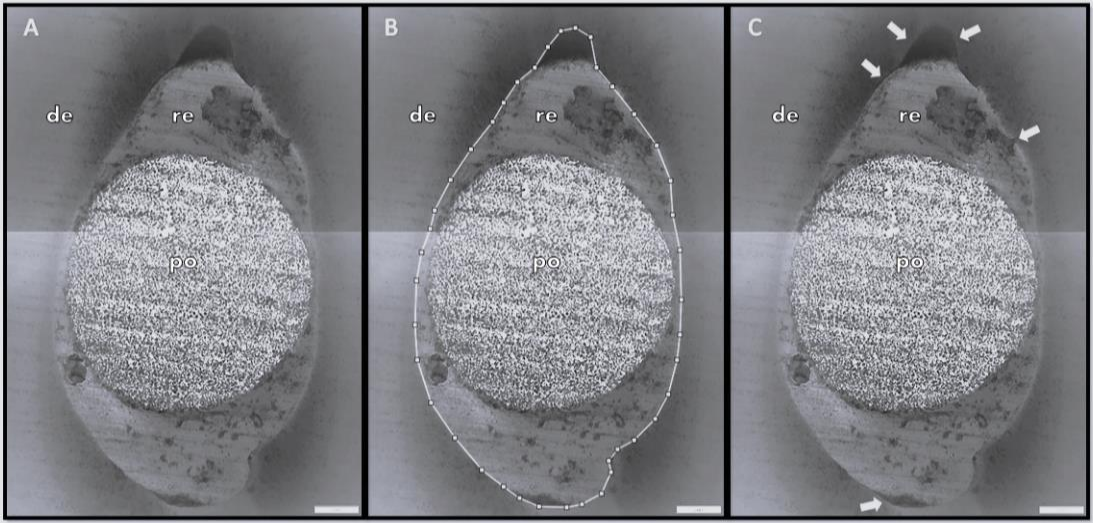
Representative image obtained under CLSM at ×40 magnification. (A) = confocal image of resin cement/glass fiber post junction to the root dentin; (B) = measurement of the total perimeter of the adhesive interface; and (C) = evident gaps (arrows) at the interfacial adaptation. (po = post; re = resin cement; de = dentin)

The first slice of the middle third was analyzed by confocal laser scanning microscopy (CLSM) (FV1000; Olympus, Tokyo, Japan). A metallographic preparation was previously carried out with sandpaper of decreasing grit size (up to 1200) and felt discs with polishing paste. The slices were submitted to an ultrasonic bath for 5 minutes, rinsed in distilled water and decalcified superficially with 37% phosphoric acid for 15 seconds ^17^. Two images in stitching mode at ×40 magnification were obtained from the cervical surface and transformed into a single one, with a size of 1376 x 1038 pixels and a scale set to 1 mm. In addition, a mapping of the images in the CLSM software itself at ×200 magnification was performed to assist in the identification of gaps. The method proposed by De-Deus et al. ^(^ ^18^ to calculate the presence of gaps between the filling material and the dentin was adapted. First, in each sample, the total perimeter of the cement/dentin interface was measured. Then, the perimeter with gaps at the cement/dentin interface was measured. The percentage of gaps was calculated by the ratio between the total perimeter and the perimeter with gaps (Figure 1). A blinded observer was responsible for this analysis. All computational work was performed using Image J software (National Institutes of Health, Bethesda, USA). The calibration consisted of repeating the analysis of gaps of 10 slices, with an interval of two weeks. Examiner reproducibility, which was calculated using the intraclass correlation coefficient, was 0.997.

### Statistical analysis

After the Shapiro-Wilk and Levene tests, the bond strength values presented a normal homoscedastic distribution, but the gap values did not. Based on that, bond strength data were analyzed by two-way ANOVA and Tukey’s post-hoc tests. For data on the gaps, post-hoc pairwise comparisons were performed using the Kruskal-Wallis test adjusted using the Bonferroni method. The chi-square test was used to analyze the failure modes. The level of statistical significance was set at p < 0.05. All analyses were performed using the SPSS Statistics V.26 program (SPPS Inc., Chicago, USA).

## Results

### Push-out bond strength

**Figure 2:**
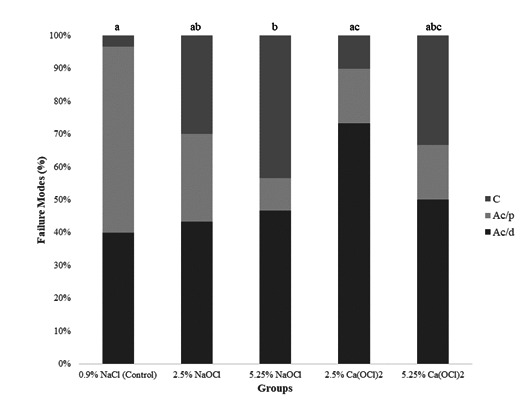
Failure modes (%) in each group after push-out. Different lowercase letters represent significant differences between groups (p <0 .05). Ac/d = mainly adhesive failure at cement/dentin interface; Ac/p = mainly adhesive failure at cement/post interface; C = cohesive failures of the dentin.

The mean and standard deviation of bond strength are shown in Table 1. The type of irrigant (p < 0.05) and the third of the post space (p < 0.001) had a significant effect on the bond strength. 5.25% NaOCl was associated with the lowest bond strength values when compared to the control (p < 0.05). Regardless of concentration, Ca(OCl)_2_ had no negative influence on the bond strength (p > 0.05). Bond strength decreased from coronal to apical in the post space (p < 0.001). The failure modes are shown in Figure 2. 5.25% NaOCl generated more cohesive failures compared to the control group and Ca(OCl)_2_ 2.5% (p < 0.001).

**Table 1:** Mean and standard deviation of bond strength values (MPa) in the root dentin of the post space in relation to different irrigating solutions and root thirds

Groups	Intraradicular Tooth Region			
	Cervical	Middle	Apical	Total
0.9% NaCl (control)	9.72 ± 3.72	7.55 ± 4.35	4.22 ± 3.75	7.16 ± 4.45^a^
2.5% NaOCl	7.40 ± 1.76	4.85 ± 2.56	3.39 ± 2.09	5.21 ± 2.68^ab^
5.25% NaOCl	5.85 ± 3.62	4.41 ± 3.03	3.28 ± 2.58	4.51 ± 3.18^b^
2.5% Ca(OCl)_2_	7.57 ± 1.74	4.77 ± 3.22	3.02 ± 1.69	5.12 ± 2.95^ab^
5.25% Ca(OCl)_2_	6.78 ± 2.31	4.85 ± 4.24	3.66 ± 3.38	5.09 ± 3.53^ab^
Total	7.46 ± 2.95^A^	5.29 ± 3.59^B^	3.51 ± 2.72^C^	

NaCl, sodium chloride; NaOCl, sodium hypochlorite; Ca(OCl)_2_, calcium hypochloride

Different lowercase letters in column mean statistically significant difference between irrigation solutions (p < 0.05). Different uppercases letters in row mean statistically significant difference between thirds (p < 0.05)

### Evaluation of adaptation

The median and percentile values in the percentage of gaps at the cement/dentin interface are shown in Table 2. The groups treated with NaOCl showed the highest percentage of gaps (p < 0.05) and the groups treated with Ca(OCl)_2_ had no influence on the occurrence of gaps, regardless of the concentration (p > 0.05), when compared to the control.

**Table 2:** Gaps values in percentage (Median [P25-P75]) of the cement/dentin interface of the post space in relation to different irrigating solutions

Groups	Gaps
0.9% NaCl (control)	12.99 (7.84-16.50)^a^
2.5% NaOCl	24.13 (18.05-45.38)^b^
5.25% NaOCl	24.49 (20.23-46.62)^b^
2.5% Ca(OCl)_2_	20.87 (8.56-30.82)^ab^
5.25% Ca(OCl)_2_	18.03 (13.05-31.94)^ab^

NaCl, sodium chloride; NaOCl, sodium hypochlorite; Ca(OCl)_2_, calcium hypochloride

Different lowercase letters represent significant differences between groups (p < 0.05)

## Discussion

The present study found lower bond strength values and a greater presence of gaps when 5.25% NaOCl was used compared to the control. In this sense, 2.5% NaOCl was also associated with higher percentages of gaps. The cervical third of the post space was associated with greater bond strength. Thus, the null hypotheses were rejected.

Lower bond strength in the post space for higher concentrations of NaOCl was also reported by other studies ^5^ ^,^ ^7^. NaOCl interacts with the organic portion of dentin causing collagen degradation. Furthermore, the higher the concentration of NaOCl, the greater the collagen degradation ^(^ ^19^. Therefore, since bond strength is directly related to the quality of the dentin substrate, these facts explain the findings of this study. The group treated with 5.25% NaOCl had more cohesive failures compared to the control and 2.5% Ca(OCl)_2_ groups. A possible explanation is based on the fact that NaOCl is associated with lower values of fracture resistance ^(^ ^11^, microhardness, and flexural strength of the dentin ^(^ ^10^, facilitating cohesive failures.

In this study, the third of the post space had a significant impact on bond strength. The values followed the decreasing sequence: cervical > middle > apical. These results are possibly attributed to the fact that the number of dentinal tubules per mm^2^ decreases from the coronal portion of the root canal to the apical one, therefore, decreasing the density of the hybrid layer in the same direction, and the adhesion becoming poorer ^(^ ^20^. Another point to consider is the light transmission/reach difficulty at deeper layers, which despite herein it being used as a dual-curing agent, it is known that such aspect may alter the degree of conversion of the resin material and consequently is logical to assume that the deeper the region, the lower bond strength expected ^(^ ^21^.

During fiber post luting, polymerization shrinkage, bubbles, and gaps negatively affect retention ^(^ ^22^. Although premolars may present a complex internal anatomy, equivalent degrees of adaptation are reported between oval and circular posts even in the presence of oval root canals, since the associated resin cement occupies the unfilled areas ^23^. Despite the cement application method in the post space can influence the formation of gaps ^24^, the same luting technique was used in all groups, and herein the evaluation of gaps was made by a researcher that was blind for the study group being accessed. Therefore, it is consistent to assume that in our results the irrigation solution really influenced the formation of gaps, as it was observed statistical differences among conditions. Furthermore, a previous systematic review reported that the simultaneous application of cement around the post and inside the root canal is associated with less adhesion compared with the application only around the post ^25^, being therefore, the second method chosen for use in this study. In our results, the presence of gaps at the cement/dentin interface was more prevalent in the groups treated with NaOCl, regardless of concentration, compared to the control. Changes in dentin structure caused by the use of NaOCl and the consequent impact on adhesion are the possible reasons for this finding ^(^ ^19^. The use of Ca(OCl)_2_ did not influence bond strength and the occurrence of gaps, possibly due to its compatibility with the dentin not altering its main properties ^(^ ^10^. Recent studies have used CLSM to assess the presence of gaps at the cement/dentin interface ^(^ ^26^ ^,^ ^27^. The use of CLSM is an accurate, non-destructive method, with results comparable to scanning electron microscopic analysis ^(^ ^28^.

In this study, we chose to use a self-etching adhesive system and dual-curing resin cement. Self-etching adhesives have results comparable to conventional adhesives, in terms of continuity of the hybrid layer and presence of resin tags. However, they are highly recommended when dentin is the main tissue to be bonded due to less critical steps when compared to etch and rinse systems ^(^ ^29^. Dentin presents lower mineral content than enamel and based on that the benefits of phosphoric acid etching use become questionable ^(^ ^30^. Dual-curing resin cements are associated with greater bond strength than self-adhesive cements ^(^ ^31^.

Previous studies evaluated the influence of Ca(OCl)_2_ on the adhesiveness of the post space using a self-adhesive cement ^(^ ^12^ ^,^ ^13^, or a conventional adhesive system and a dual-curing cement ^(^ ^14^. In such sense, discussion and a direct comparison of the findings should be done with caution, as such different luting systems present distinct mechanisms of interaction with the dentin, thus is logical to assume that the solution may present different performance depending on such variations. Based on that, more studies are still required to completely understand and validate the mechanisms of interaction between such solutions and the different existing luting systems.

The use of only one diameter of a stainless-steel plunger for all post-thirds during the push-out test may be pointed as a limitation of the present study. Although we used a standard diameter of a plunger for all slices and groups, which provided coverage of the post without touching the dentin wall, the root canal has a very complex internal configuration and different internal diameters according to its root third. Therefore, customizing a plunger of different diameters for each third may be a more suitable strategy to better represent bond strength values ^32^. In addition, another limitation of our study was that we did not assess bond strength longevity. The bonding behavior can be different through time, with the aging of the samples decreasing the bond strength ^33^. Thus, different aging conditions should be investigated in further studies to observe if the negative effect of NaOCl would be even more pronounced.

In our findings, 2.5% NaOCl did not change the bond strength. However, it is associated with higher percentages of gaps. Gaps will act as defects and stress concentrator zones during teeth function, based on that, with time, it may predispose to failures of the restorative set. On the other hand, Ca(OCl)_2_ does not change the bond strength and is not associated with the occurrence of gaps, regardless of its concentration (2.5% or 5.25%), corroborating such solution as a promising alternative to be used on such scenario. Summed to that, from an antimicrobial point of view, NaOCl and Ca(OCl)_2_ have similar effects, and the higher the concentration of both, the greater their antiseptic action ^(^ ^9^. Assuming that an ideal solution for use in the post space must present a balance between its antimicrobial capacity and its influence on dentin adhesiveness, 5.25% Ca(OCl)_2_ seems to be the best option when using a dual-curing self-etching resin cement system. To consolidate the findings, more studies are needed to evaluate the different resin cements, as well as to carry out well-designed randomized clinical trials to investigate the clinical behavior of 5.25% Ca(OCl)_2_ in the success rates of fiber post adhesive luting.

Considering the limitations of the in vitro study, in cases where a restorative plan includes a fiber post-luted with dual-curing resin cement, Ca(OCl)_2_ is a good option to decontaminate the post space without negatively impacting dentin adhesion.
